# Examination of Raw Samples and Ethanol Extracts of Gerês Propolis Collected in Different Years

**DOI:** 10.3390/plants12223909

**Published:** 2023-11-20

**Authors:** Ana Rita Caetano, Rafaela Dias Oliveira, Rui Filipe Cerqueira Pereira, Tiago Vidal Cardoso, Andreia Cardoso, Cristina Almeida-Aguiar

**Affiliations:** 1 Department of Biology, School of Sciences, University of Minho, Campus of Gualtar, 4710-057 Braga, Portugal; ritacaetano98@gmail.com (A.R.C.); rafaeladiasoliveira98@gmail.com (R.D.O.); pg46659@alunos.uminho.pt (R.F.C.P.); tiaago.98@gmail.com (T.V.C.); candreiacardoso@gmail.com (A.C.); 2 Centre of Molecular and Environmental Biology (CBMA), University of Minho, Campus of Gualtar, 4710-057 Braga, Portugal; 3 Life and Health Sciences Research Institute (ICVS), School of Medicine, University of Minho, Campus of Gualtar, 4710-057 Braga, Portugal; 4 ICVS/3B’s-PT Government Associate Laboratory, 4710-057 Braga, Portugal

**Keywords:** Portuguese propolis, raw samples, quality parameters, ethanol extracts, chemical characterization, antimicrobial activity, antioxidant activity

## Abstract

Propolis, a natural resin created by bees, has garnered significant attention from both the scientific community and industry due to an impressive range of bioactivities. Nonetheless, the intrinsic variability in its chemical composition and bioactive profiles has been hindering propolis’ full potential use. We previously showed that ethanol extracts (EEs) of a Portuguese propolis sample (Gerês) collected over four consecutive years displayed similar chemical and biological profiles, a constancy never documented before. However, the characteristics of the unprocessed samples of Gerês propolis were never described. Hence, the central objective of this study is to assess the quality parameters of unprocessed propolis samples collected from Gerês (G), over a four-year period (2019–2022), alongside the analysis of the chemical composition and bioactivities of the EEs prepared with the same raw samples. The ash, wax, balsam and water contents of the unprocessed samples—G19 to G22—showed minor fluctuations, likely attributed to uncontrollable natural events impacting the propolis source and collection process. On the other hand, the antimicrobial and antioxidant activities of all the four ethanol extracts (G19.EE–G22.EE) consistently align with prior studies. Furthermore, the Gerês propolis extracts showed remarkable uniformity in chemical composition parameters too, particularly concerning total polyphenol, flavonoid and ortho-diphenol contents. In summary, our research reinforces the beneficial properties of propolis and show that extracts’ bioactivities remain within the reference ranges for Gerês propolis, despite minor differences in unprocessed samples, suggesting a consistent action over time. Thus, this work could be instrumental towards the establishment of standard parameters for propolis applications, offering valuable insights to this field of propolis research.

## 1. Introduction

Propolis is a resinous aromatic material produced by honeybees through the combination of resin from various plant buds and exudates with beeswax, pollen and bees’ salivary enzymes [1,2]. Bees use propolis to seal the gaps in their hives, protect themselves from diseases and maintain the health of their colony [3]. Thus, it makes sense the Greek origin of the word, meaning pro (in defence) and polis (city), that is, in defense of the city or the hive, in this particular case [4].

Propolis has a highly variable chemical composition due to specific biological and environmental factors, such as the bee species that produce this natural mixture, the type of vegetation around the hive and the geographical and climatic conditions [5,6,7]. Different types of this beehive product are currently recognized based on their geographical origin and botanical sources [1]: in Europe, North America and some regions of Asia and New Zealand, the main plant source of the resin is *Populus* spp., with this type of propolis being known as Poplar [5]; in Brazil, four propolis types are known, specifically Green, Red, Clusia and Brown; in Russia, the principal type is Birch propolis [5]; and in the Mediterranean area, plant sources are *Crupessus* spp. and *Conifer* spp. [1], while the main resin source in African propolis is *Macaranga schweinfurthii.* Australian propolis has *Acacia paradoxa* and *Corymbia torelliana* trees as the principal sources of resin and Mangifera propolis is a propolis type found in tropical regions where the plant source is *Mangifera indica* [1]. According to the most recent literature [8], approximately 800 compounds have been identified in different types of propolis, a number expected to increase even further as research progresses. However, despite the great diversity of compounds, propolis is generally composed of 50% resin, 30% wax, 10% essential oils, 5% pollen and 5% of other substances, which include minerals and organic compounds [9].

After honeybee domestication, humans began to explore beehive products for their own benefit. Propolis, or bee glue, is a very popular natural remedy that has been used for centuries due to its outstanding medicinal properties and remains popular nowadays [10,11]. However, the propolis complex and variable chemical composition has hampered a more widespread usage of this natural product, particularly within the medical community [3]. In fact, the literature is full of publications describing factors influencing propolis composition, and the existence of different types of propolis with distinct and unique chemical profiles has been reported [1,12], making the standardization and quality control one of the main challenges in propolis research [3,8]. In Portugal and Europe, there is still no regulation for propolis standardization despite the efforts of local and international commissions working on the quality of beehive products. Even so, according to the International Honey Commission, a complete analysis should evaluate parameters that can be accepted as universal, such as balsamic, ash, wax and water contents, as well as the presence or absence of impurities [13]. Furthermore, the active compounds—specifically phenolic acids and flavonoids—should be known, and their contents should be high [14,15,16]. On the other hand, in Brazil, where the study of propolis is well established, the Technical Regulation of Propolis Identity and Quality (TRPIQ) of the Ministry of Agriculture, Livestock and Food Supply has already established the minimum quality requirements for Brazilian propolis parameters in order to be available for national or international commercialization (ash content ≤ 5%; water content ≤ 8%; wax ≤ 25%) [17].

Portuguese propolis characteristics have been explored, with researchers being focused on the chemical and biological characterization of samples from different regions of the country [14,18,19,20,21,22,23,24,25,26,27,28,29,30,31]. Following this research trend on Portuguese propolis, some studies emphasized the interesting properties of propolis from Gerês, namely an outstanding antioxidant activity [25,28] and the widely known antibacterial and antifungal activities [25,28,30], as well as an anti-inflammatory [26] and anticancer potential [19,22]. Moreover, ethanol extracts of propolis from Gerês have shown unique and consistent biological and chemical profiles over the years [25], holding great potential for establishing standardized criteria for the assessment of Portuguese and Poplar propolis quality. However, despite the extensive research on the biological and chemical aspects of these propolis extracts [19,22,26,30,31], comprehensive research regarding the raw/unprocessed propolis samples is currently missing. Considering this, our study aims to evaluate the quality parameters of unprocessed samples collected from Gerês over four consecutive years (2019–2022) and determine their impact on the chemical composition, antioxidant capacity and antimicrobial activity of the ethanol extracts (G.EEs) derived from the same raw samples.

## 2. Results

### 2.1. Quality of Raw Propolis Samples

The quality of propolis samples (G19–G22) was evaluated by determining their ash, water, wax and balsam contents (Table 1).

### 2.2. Chemical Characterization of the Ethanol Extracts of Propolis

The total polyphenol (TPC), the total flavonoid (TFC) and the total ortho-diphenol (TOC) contents were determined for the four G.EEs in order to provide a general chemical characterization of each extract (Table 2). The TPC values varied between 107.96 ± 5.6 and 226.73 ± 4.3 mg GAE/g of extract. The values obtained for TFC ranged from 31.9 ± 1.1 to 40.94 ± 1.32 mg QE/g of extract. Finally, the calculated TOC values were between 272.3 ± 4.7 and 332.27 ± 9.58 mg GAE/g of extract.

### 2.3. Antioxidant Activity of the Ethanol Extracts of Propolis

The methodology based on the reduction of a stable free radical, the DPPH•, was used to assess G.EEs ability to scavenge free radicals. The values of EC_50_ (a concentration that produces half of the maximal response) ranged from 13.37 ± 0.38 μg/mL to 21.49 ± 1.15 μg/mL (Table 3).

In addition to the DPPH• scavenging assay, the 2,2′-azino-bis (3-ethylbenzothiazoline-6-sulfonic acid) (ABTS) assay was also used to evaluate the antioxidant capacity of the same propolis extracts. ABTS assay employs the absorption of the ABTS• colored radical cation to measure a compound’s antioxidant ability. Trolox was employed as the standard, and the EC_50_ values ranged between 8.38 ± 0.25 and 10.53 ± 0.18 μg/mL (Table 3). Radical scavenging activity was observed in all tested G.EEs using both methodologies.

### 2.4. Antimicrobial Activity of the Ethanol Extracts of Propolis

Antimicrobial activity is one of the most documented and widely studied propolis bioactivities. The minimum inhibitory concentration (MIC) values of the four prepared G.EEs were determined against a panel of selected bacteria and yeast susceptibility indicator strains (Table 4).

## 3. Discussion

Quality parameters play a pivotal role in assessing the composition and purity of raw propolis samples, from which valuable bioactive substances are extracted [32]. The chemical richness and intricacy of propolis is a major challenge for the standardization of such criteria; but the establishment of standardized quality parameters is imperative to ensure the suitability of propolis for a diverse array of applications, namely within the health and industrial sectors. In the absence of universally recognized national or international standards and regulations, the evaluation of propolis quality routinely centers on the scrutiny of four specific parameters: ash, water, wax and balsamic contents [13,33]. Currently recognized as quality benchmarks, these parameters have established reference values that make it possible to gauge propolis’ overall quality and purity for several purposes [13,17,21].

All the studied Gerês propolis samples exhibited ash and wax levels below the maximum values specified in the literature for Portuguese/European propolis (2% and 25%, respectively) [21], as well as those permitted by the Technical Regulation of Propolis Identity and Quality (TRPIQ) (5% for ash and 25% for wax contents) [17]. This is a desirable condition since a higher ash content may suggest the presence of inorganic impurities or contamination [34]. In addition, high levels of waxes can adversely affect propolis’ purity and quality and could indicate poor processing or contamination [35,36]. Regarding the water content, the unprocessed samples displayed slightly higher values than those reported in the literature for Portuguese propolis (≤5%), but within the values validated by the TRPIQ (≤8%). G21 exhibited a significantly lower water content (2.42 ± 0.14%) compared to the other samples, while G19, G20 and G22 presented similar contents (values ranging from 6.23 ± 0.02% to 6.56 ± 0.19%). This parameter can be influenced by factors like the handling and storage time, as documented by [21]. In fact, the slightly higher water content in these samples can be explained by the storage time since this parameter was only evaluated in G19, G20 and G22 after a couple of years of its storage at 4 °C, while G21 was analyzed in the year of its collection. Balsamic content is one of the most important indicators of propolis quality [37,38]. The four raw samples of propolis from Gerês exhibited high levels of balsamic content and mostly fulfilled the minimum content established for European propolis (≥45%). Specifically, G20 exhibited the highest balsamic content (55.00 ± 8.66%), followed by G21 (51.67 ± 2.89%) and G22 (48.33 ± 3.54%), while G19 displayed the lowest content (44.43 ± 2.83%). Yet, significant differences between the four propolis samples were not observed. These findings indicate that propolis samples from Gerês meet the quality parameters established by the literature [13,21] and the available Brazilian regulation (TRPIQ) [17] regarding ash, water, wax and balsamic contents. Furthermore, and notably, the values obtained within these quality criteria exhibit a remarkable level of consistency over the four-year period, highlighting the uniqueness of Gerês propolis regarding its constancy over time, a characteristic undoubtedly sustained by the organic beekeeping methods employed in the apiary as well as its strategic localization within the protected and controlled confines of Gerês—the National Peneda-Gerês Park.

The pharmacological properties of propolis have often been attributed to its phenolic compounds [39,40]. Thus, to characterize a propolis sample extract and determine its quality/value, it is common to assess its phenolic content [13]. Spectrophotometric methods that allow a simple and quite fast phenolic content evaluation were used herein, instead of other methodologies like gas chromatography (GC) or high-performance liquid chromatography (HPLC) that allow a more in-depth study. However, such spectrophotometric protocols have been recently validated for propolis by an inter-laboratory study conducted by the International Honey Commission in order to harmonize analytical methods and assess their accuracy [41]. The four G.EEs presented some differences in their phenolic composition (Table 2), which can be explained by the fact that, in distinct years, the flora visited by the bees was different. Another characteristic of Gerês propolis that may explain some of the variations observed is that it results from the mixture of propolis collected from different locations/places of the apiary, and while the origin was consistent for G19, G21 and G22 (Roca + Toutelo + Felgueiras), G20 lacked one of those places (Toutelo + Felgueiras). Differences in propolis harvesting locations can be related to the occurrence of uncontrollable natural events in those particular areas, namely a wildfire that led to the extinction of one of the original places (Amadeu Fortunas, personal communication). Among the four studied extracts, G20.EE exhibited the highest value of TPC (226.73 ± 4.3 mg GAE/g extract) and the highest balsamic content as well (Table 1), which could suggest that it is the most active extract. Indeed, G20.EE displays the highest DPPH scavenging activity (Table 3). G21.EE and G22.EE presented the most prominent TFC values. Considering the link between this bioactivity and flavonoids [31,42,43], it is feasible to predict a higher antibacterial activity for these two extracts, which was also confirmed (Table 4). Concerning the TOC values, only G21.EE is significantly different from the remaining extracts.

The TPC values obtained for G.EEs (107.96 ± 5.6 to 226.73 ± 4.3 mg GAE/g extract) are slightly different from the results of Gerês propolis extracts from earlier years (2011–2016; 143.0 ± 1.9 to 224.60 ± 10.86 mg GAE/g), yet in the same range. Compared to values obtained for other regions of Portugal, a propolis sample from the northeast of the country showed a TPC of 329.00 ± 0.01 mg GAE/g of extract, considerably higher than the values described for the four tested G.EEs. A sample from the central region displayed 151.00 ± 0.01 mg GAE/g of extract [18] and a sample of Beira Alta exhibited 160.40 ± 16.56 mg GAE/g of extract [23], whereas 150.39 ± 6.29 mg GAE/g of extract was the TPC reported for an ethanol extract of propolis from Caramulo (Central region) [27], a result more similar to the TPC values of G.EEs. Regarding the flavonoid content, the evaluated G.EEs presented a TFC between 31.9 ± 1.1 and 40.94 ± 1.32 mg QE/g of extract, similar to the values obtained for propolis from Gerês in previous years [28] (31.0 ± 1.3 to 51.7 ± 0.9 mg QE/g of extract; ns, *p =* 0.8645)). However, the TFC of G.EEs appears to be lower than the results described for an ethanol extract of propolis from Caramulo (65.44 ± 1.61 mg QE/g of extract; [27]) and for ethanol extracts of propolis collected in different years (2011 to 2015) from the Guarda region (TFC ranging from 44.7 ± 12.0 to 101.8 ± 4.2 mg QE/g) [29]. Although samples are from the same country, variations among propolis from distinct regions can be observed since their content depends on the type of flora around the hives. As a result, different propolis samples can present distinct compounds and proportions, with G.EEs actually being poorer in flavonoids content. Even so, the TFCs of G.EEs (Table 3) are within the range of values determined for several locations in Portugal (5.2 ± 0.3 to 114.2 ± 0.1 mg/g of extract [21]). On the other hand, a high concentration of phenolic compounds has been linked to a strong antioxidant action [44]. Therefore, the TPC and TFC values presented by Gerês propolis indicate a promising antioxidant activity for these extracts. Ortho-diphenols are a recently evaluated parameter in propolis [34]; though, they are extremely relevant in light of the correlation that appears to exist between their presence and the antioxidant potential of natural products [45,46]. This correlation seems to be valid also in the case of Gerês propolis [31]. Considering all the tested extracts, G21.EE has the highest ortho-diphenol content value (Table 3), and it is apparently more similar to the TOC value obtained for an ethanol extract of Caramulo propolis (314.17 ± 13.09 mg GAE/g) by [27]. The main phenolic compounds of Gerês propolis have been previously identified via HPLC-DAD-ESI/MS^n^ analysis, using samples collected between 2011 and 2015, and showed to be apigenin, pinobanksin, chrysin, acacetin, galangin, kaempferide, kaempferol, caffeic acid, caffeic acid isoprenyl ester (CAIE), 3,4-dimethyl-caffeic acid (DMCA), p-coumaric acid, p-coumaric acid methyl ester and ferulic acid [19,25,31]. These phenolic constituents remained consistent throughout the five-year period, with just subtle variations observed in their abundance. Considering this and the previously reported constancy of bioactive and chemical profiles of G11.EE to G14.EE by Freitas et al. [25], as well as the relatively consistent bioactivities profiles of the studied G.EEs herein, it seems reasonable to anticipate that G19.EE–G22.EE could also have a similar phenolic composition.

After analyzing the quality parameters of G19–G22 unprocessed samples and assessing the chemical characteristics of the respective ethanol extracts, we shifted our focus to propolis’ biological features, namely its antimicrobial and antioxidant capacities. According to Sheng et al. [47], a natural product can be classified as a possible natural antioxidant if it exhibits DPPH• and ABTS• radical scavenging capacities, and previous studies reported Portuguese propolis on numerous occasions as a natural product with a high antioxidant capacity [18,25,48]. Falcão et al. [21] described that ethanol extracts of propolis samples collected from Portuguese northern and coastal regions displayed EC_50_ values ranging from 10 to 30 μg/mL, values notably and significantly lower compared to those reported for samples from other areas of Portugal. Particularly, the sample collected in Montalegre exhibited the lowest EC_50_ value (10 μg/mL), indicating a superior antioxidant activity. Subsequently, Freitas and co-authors [25] showed that the ethanol extracts of Gerês propolis collected over a span of four consecutive years (2011 to 2014) exhibited EC_50_ values ranging from 14.41 ± 0.56 to 25.24 ± 2.45 μg/mL (DPPH• assay). In the present work, the EC_50_ values for the G.EEs varied between a similar range of 10.85 ± 0.18 μg/mL to 21.49 ± 1.15 μg/mL (DPPH• assay) and 8.38 ± 0.25 μg/mL to 10.53 ± 0.18 μg/mL (ABTS• assay). G21.EE and G22.EE exhibited the lowest EC_50_ values among the tested extracts, in both scavenging assays, pointing to a higher antioxidant potential. The observed differences in the balsamic content and phenolic composition of the extracts, as mentioned earlier, can provide an explanation for such results. Although there were variations in the EC_50_ values among the extracts, it is evident that Gerês propolis, as a whole, consistently retained its antioxidant activity over the twelve years it was studied. This finding highlights the remarkable preservation of this property across extracts with variable phenolic compositions, emphasizing the robust and potent nature of Gerês propolis in effectively neutralizing free radicals. Lastly, taking a broader perspective into consideration, the EC_50_ values of Gerês propolis are significantly lower than those documented for propolis samples worldwide [49,50], indicating a heightened antioxidant capacity. As an outcome, Portuguese propolis from Gerês can be classified as a promising and potent antioxidant product, demonstrating consistent antioxidant activity. Indeed, the notable disparities in EC_50_ values between Gerês propolis and global propolis samples highlight the improved antioxidant potential of Gerês propolis, reinforcing its position as a valuable source of natural antioxidants.

Antimicrobial activity is one of the most studied propolis bioactivities, especially at a time when the high resistance of some microorganisms to antibiotics and antifungals is becoming a global threat [51,52,53]. Indeed, the study of this biological property can lead to the identification of innovative approaches to combat the worldwide challenge of antimicrobial resistance, either by the use of propolis extracts or its compounds and/or by its combination with antibiotics or other natural products [30,54]. The antimicrobial properties of propolis from Gerês have been studied by our research group for years. Overall, our data demonstrated that Gram-negative bacteria, like *E. coli*, are less susceptible to the action of propolis than Gram-positive bacteria (Table 4), a result supported by many other studies [5,55,56,57,58,59,60,61,62,63,64,65,66,67] and explained by the structural differences in the bacterial cell walls [64]. Beyond *E. coli*, MRSA was also resistant to all G.EEs at the tested concentrations (Table 4). On the contrary, all G.EEs showed some activity against *Staphylococcus aureus* (Table 4), yet this activity seems to be year-dependent since MIC values of G.EEs (2019–2022) ranged from 200 to 750 µg/mL. A red propolis extract was shown to be more active against MRSA (MIC = 50 µg/mL) than against *S. aureus* (MIC = 100 µg/mL) [68], while a Brazilian propolis extract showed similar efficacy against the same two strains (MIC = 1.420 µg/mL) [69]. G20.EE is the extract with the narrowest antibacterial spectrum, being the one with the highest MIC (750 µg/mL) against *P. acnes.* Extracts from subsequent years—G21.EE and G22.EE—display lower MIC values against this pathogen (200 µg/mL) than extracts from previous years, namely G17.EE, G18.EE and G19.EE (MIC = 500 µg/mL). One of the most unique and main biological characteristics of Gerês propolis is its efficacy against the Gram-positive spore forming bacteria of the genus *Bacillus,* with MIC values between 50 and 100 µg/mL, regardless of the harvesting year. Given that this antimicrobial activity has been consistently reported for over two decades, it could be considered an indicator of the similarity of samples obtained in different years.

Ethanol extracts of propolis from Gerês are not effective in inhibiting the growth of yeast, namely *S. cerevisiae* and *C. albicans*. In fact, only G21.EE showed antifungal activity against both yeast (MIC = 2000 µg/mL), and G19.EE showed the lowest MIC (1500 µg/mL) against *C. albicans*. Beyond the G.EEs studied herein, the ethanol extracts of samples collected in the same region from 2011 until 2015 [25,28] support the fact that G.EEs have little antifungal activity. This seems to be different from what has been observed for the same propolis type (Poplar type) from different European regions: for instance, a Spanish propolis ethanol extract inhibited the growth of *S. cerevisiae* at lower concentrations than G19.EE and G21.EE (MIC values between 500 and 1500 µg/mL) [70], and a MIC value of 31.25 µg/mL was reported for a French propolis against *C. albicans* [71].

## 4. Materials and Methods

### 4.1. Propolis Samples: Origin and Quality Analysis

The four propolis samples employed in this research were harvested during a four-year period (2019–2022) from an apiary named Gerês, as it is situated near the village of Montalegre, in Gerês, Portugal (41°45′41.62″ N; 7°58′03.34″ W). Gerês is a region in the northern part of the country that houses the Peneda-Gerês National Park, a protected area where the soil, water, flora, fauna and overall landscape are preserved. Typically, propolis from Gerês is obtained by blending propolis collected from plastic traps/grids (Bee Equipment, Prague, Czech Republic) placed in beehives distributed across different locations within the apiary (specifically the places Roca, Toutelo and Felgueiras, in the context of the four samples under study). Each Gerês propolis sample consists of a single annual collection, with harvesting taking place between August and September. Plastic propolis traps are removed from the beehives, put inside plastic bags and frozen overnight. Grids are twisted inside the bag to release the propolis. Gerês propolis is obtained by mixing in equal proportions the propolis obtained from the grids removed from Roca, Toutelo and Felgueiras beehives. Samples were designated by the capital letter G followed by the harvesting year (G19, G20, G21, G22) and were stored at 4 °C until use.

#### 4.1.1. Ash Content Analysis

The ash content was determined using a modified version of the method described by [13]. Briefly, 1 g of propolis [A1] was added to a pre-weighed crucible [A2] and incinerated in a muffle furnace at 550 °C for 3 h until ash was obtained. Upon cooling of the muffle furnace, the ash-containing crucible was placed in a desiccator overnight and reweighed [A3] the following day. Ash content was expressed as a percentage (%, *m/m*) and calculated from the expression [(A3 − A2)/A1 × 100)].

#### 4.1.2. Water Content Analysis

Water content was determined as described by [72]. A previously weighed beaker containing 4 g of propolis [H0] was covered with aluminum foil and left in an oven at 105 °C for 5 h. After drying, the beaker was placed in a desiccator overnight to cool and was reweighed [H1]. The water content was expressed as a percentage (%, *m/m*) and calculated from the expression [(H0 − H1)/4 × 100)].

#### 4.1.3. Wax Content Analysis

Wax content was determined using the method of [73], which relies on the specific density difference between propolis and beeswax. For this, 1 g of propolis [W1] was combined with 2.5 mL of deionized water and this mixture was cautiously heated in a microwave oven, followed by cooling to room temperature (RT) until three distinct layers formed. The wax-top layer was carefully removed, weighed [W2] and the wax content was calculated from the expression [W2/W1 × 100)] andexpressed as a percentage (%, *m/m*).

#### 4.1.4. Balsam Content Analysis

The balsamic content was determined as described by [74], by adding 15 mL of 70% ethanol (Fisher Chemical, Hampton, NH, USA) to 0.5 g of raw propolis [B1]. The mixture was incubated at 30 °C with agitation at 125 rpm for 24 h, filtered, and the filtrate was stored at 4 °C. The residue was re-extracted under the same conditions. After a second filtration, filtrates were combined and ethanol 70% was added to a final volume of 50 mL. To calculate the balsamic content, aliquots of 2 mL were taken from this mixture and the solvent was evaporated by nitrogen flow. The dry extract was weighed [B2] and the balsamic content was calculated (B2/B1 × 100) and expressed as a percentage (%, *m/m*).

### 4.2. Propolis Extraction

Propolis ethanol extracts were prepared with absolute ethanol (Fisher Chemical, Hampton, NH, USA), as previously described [25], as the use of this solvent allowed us to obtain extracts with higher levels of bioactive compounds and wider biological properties [19,23,24,25]. Briefly, raw samples were incubated with absolute ethanol for 24 h, in the dark with stirring, the suspension was filtered, and the filtration residues were collected and incubated again with ethanol under the above-referred conditions, followed by another filtration. The ethanol extracts G19.EE, G20.EE, G21.EE and G22.EE were obtained after solvent evaporation, and stored at 4 °C, in the dark. Stock solutions of G.EEs were prepared for the following assays by dilution in the same extraction solvent.

### 4.3. Chemical Characterization of G.EEs

#### 4.3.1. Determination of Total Polyphenol Content (TPC)

The total polyphenol content (TPC) of G.EEs was determined using the Folin–Ciocalteu colorimetric method [75]. For this assay, 10 μL of each extract, 50 μL of Folin-C (Sigma-Aldric, St. Louis, Mo, USA, 1:10) and 40 μL de Na_2_CO_3_ (Merck, 7.5%; *w*/*v*) were added per well of a 96-well plate to obtain G.EEs concentrations from 50 to 300 μg/mL. For each concentration, a blank was prepared with each extract and ethanol. A control with Folin-C, Na_2_CO_3,_ and ethanol was also used. After 1 h incubation in the dark and at RT, the absorbance was read at 760 nm (Spectra Max Plus 384). Results were obtained via the interpolation of linear regression using gallic acid (GA) (Sigma-Aldrich, St. Louis, MO, USA) as the standard (1–30 μg/mL), and the TPC was expressed as gallic acid equivalents (mg GAE/g extract).

#### 4.3.2. Determination of Total Flavonoid Content (TFC)

The total flavonoid content (TFC) of G.EEs was determined using the aluminum chloride (AlCl_3_) colorimetric method [72]. For this assay, in 96-well plates, 50 μL of each extract and 50 μL AlCl_3_ (Acrós Organics, 2%) were added per well to obtain concentrations from 200 to 1600 μg/mL. For each concentration, a blank was prepared with each extract and ethanol. A control was also used, prepared with AlCl_3_ and ethanol. Absorbance was read at 420 nm after 1 h incubation in the dark at RT. Results were obtained via the interpolation of linear regression using quercetin (Q) as the standard (5–200 μg/mL), and TFC was expressed as quercetin equivalents (mg QE/g extract).

#### 4.3.3. Determination of Total Ortho-Diphenol Content (TOC)

The total ortho-diphenol content (TOC) of G.EEs was determined using the sodium molybdate (Na_2_MoO_4_) complexation method [76], with some modifications: 160 μL of each extract and 40 μL of Na_2_MoO_4_ (Acrós Organics, 50,000 µg/mL, prepared with 50% ethanol) were added per well of a 96-well plate to obtain extract concentrations from 25 to 300 µg/mL. A blank with each extract and ethanol 50% (*v*/*v*) was prepared for each concentration. A control was also made with Na_2_MoO_4_ and 50% ethanol. After 15 min of incubation in the dark at RT, the absorbance was read at 370 nm. Results were obtained via the interpolation of linear regression using a gallic acid calibration curve (10–200 μg/mL), and TOC was expressed as gallic acid equivalents (mg GAE/g extract).

### 4.4. Assessment of Antioxidant Potential

#### 4.4.1. DPPH Radical Scavenging Assay

The antioxidant capacity of propolis extracts was determined according to the DPPH (2,2-diphenyl-2-picrylhydrazyl) colorimetric method [25,77]. In brief, 100 µL of DPPH• working solution 0.004% (*w*/*v*) was mixed with 50 µL of each G.EE to achieve final concentrations ranging between 1 and 50 µg/mL in a 96-well plate. This plate was subsequently incubated in the dark for 20 min. Controls were prepared with ethanol and DPPH• working solution. Then, the absorbance was measured at 517 nm, using ethanol as a blank. The EC_50_ (μg/mL), corresponding to the concentration of an extract needed to scavenge 50% of the initial DPPH•, was calculated. Gallic acid was used as the standard (0.20–1.5 μg/mL; R^2^ = 0.9899) for this experiment.

#### 4.4.2. ABTS Radical Scavenging Assay

The propolis scavenging activity was also assessed using the 2,2′-azino-bis(3-ethylbenzothiazoline-6-sulfonic acid) (ABTS) cation radical decolorization assay [78,79]. G19.EE to G22.EE were diluted in absolute ethanol to yield concentrations between 0.5 and 25 µg/mL. The ABTS• working solution was prepared by mixing 7 mM ABTS aqueous solution with 140 mM potassium persulphate and subsequent incubation for 14 h to 17 h in the dark. After that, this ABTS• solution was diluted in 100% ethanol, yielding a 734 nm absorbance of 0.70. Then, 2.5 µL of each propolis dilution was mixed with 247.5 µL of ABTS• working reagent and incubated for 30 min in the dark. Absorbance was measured at 734 nm, using ethanol absolute as a blank, and EC_50_ was calculated. Trolox (0.5–10 μg/mL; R^2^ = 0.9928) was used as the standard.

### 4.5. Assessment of Antimicrobial Activity

#### 4.5.1. Strains, Media and Growth Conditions

Gram-negative bacterium *Escherichia coli* CECT 423, six Gram-positive bacteria—*Bacillus subtilis* 48886, *Bacillus cereus* ATCC 7064, *Bacillus megaterium* 932, *Propionibacterium acnes* H60803(2961351), Methicillin Sensitive *Staphylococcus aureus* ATCC 6538 (MSSA) and Methicillin Resistant *Staphylococcus aureus* M746665 (MRSA)—and the yeast *Saccharomyces cerevisiae* BY4741 and *Candida albicans* 53B, all from the microbial collection of the Department of Biology of the University of Minho, were used in this study.

Yeast cell cultures were prepared in liquid YPD medium (1% *w*/*v* yeast extract; 2% *w*/*v* peptone; 2% *w*/*v* glucose) and maintained and tested in YPDA (YPD recipe with 2% *w*/*v* agar). Bacteria were cultured in LB broth (0.5% *w*/*v* yeast extract, 1% *w*/*v* tryptone, 1% *w*/*v* NaCl) and maintained and tested in LBA (LB recipe with 2% *w*/*v* agar). Bacterial and yeast cultures were obtained after incubation at 37 °C and 30 °C, respectively; with agitation at 200 rpm. Culture growth was monitored by optical density at 600 nm (OD_600_).

#### 4.5.2. Determination of Minimum Inhibitory Concentrations

The minimum inhibitory concentrations (MICs) of G.EEs were determined using an adaptation of the agar dilution method [80]. Drops of 5 µL of exponentially growing microbial cultures (OD_600_ between 0.4 and 0.6) were inoculated on solid media—LBA or YPDA if testing bacteria or yeast, respectively—with different concentrations of each G.EE (5, 10, 50, 100, 200, 500, 750, 1000, 1500 or 2000 µg/mL).

After incubation at 37 °C for 24 h for bacteria and at 30 °C for 48 h in the case of yeast, plates were observed, and the MIC values were determined as the lowest concentration for which no microbial growth was detected. Two types of controls were made, one with just the solid medium—either LBA or YPDA depending on testing bacteria or yeast, respectively—and another with the same media supplemented with solvent in the same volume as used in the plate with the highest concentration of G.EEs (2000 µg/mL).

### 4.6. Statistical Analysis

Results obtained are represented by mean and standard deviation (SD) values of a minimum of, unless otherwise stated, three independent experiments (*n* ≥ 3) with three replicates each. One-way analysis of variance (ANOVA) was used for the comparison of more than two means and Tukey’s test for multiple comparisons. The threshold used for statistical significance was *p* < 0.05, and differences considered statistically significant were noted with different letters.

## 5. Conclusions

The ash, water, wax and balsam contents of all studied propolis samples met the quality requirements described in the literature for Portuguese/European propolis and stated by the Technical Regulation of Propolis Identity and Quality (Brazilian entity) for the control of propolis quality, although showing slight variations among them. The preliminary chemical characterization of the ethanol extracts prepared from such propolis raw samples confirmed their abundance in phenolic compounds and indicated their high quality. The extracts G21.EE and G22.EE exhibited the highest total flavonoid content, and G21.EE also showed the highest TOC content among the samples evaluated. Nonetheless, these differences do not appear to exert a significant impact on the bioactivities of the respective samples. Instead, there is a notable constancy and similarity in the biological activities among the tested propolis samples from Gerês, namely when comparing them with samples harvested from 2011. The antioxidant capacity of all the G.EEs was confirmed by both the DPPH• and ABTS• radical scavenging assays. Despite noticeable variations among the extracts, these results align with previously published data, reinforcing the consistent antioxidant activity of Gerês propolis. Antimicrobial and antifungal properties of the evaluated G.EEs are consistent with those reported in the literature for most propolis samples, namely, the high susceptibility of Gram-positive bacteria and a less pronounced anti-yeast activity, similar to the activity of G.EEs previously studied by our group, further confirming the reliability of Gerês propolis. Yet, the particularly high activity against bacteria of the genus *Bacillus* seems to be a distinctive feature of propolis from Gerês and could be used as an indicator of the similarity of samples.

In summary, our study provides evidence of the consistent behavior of Gerês propolis across different samples and years, a consistency that is prominently evident even at the unprocessed level. Moreover, the values obtained for all the tested G.EEs surpass those reported for other types of propolis worldwide. Hence, we strongly endorse further exploration of this natural product—Gerês propolis—and emphasize the importance of validating the reference quality parameters for the unprocessed samples to facilitate its commercialization and application, given its unaffected pharmacological potential.

## Figures and Tables

**Table 1:** Ash, water, wax and balsam contents of G19, G20, G21 and G22 raw propolis samples. Results are presented as mean ± SD *. Different letters mean statistically significant differences between values (*p* < 0.05).

Parameters (%, *m*/*m*)	G19	G20	G21	G22
Ash	0.84 ± 0.06	0.94 ± 0.06	1.00 ± 0.02	0.79 ± 0.16
Water	6.56 ± 0.19 ^a^	6.23 ± 0.02 ^a,b^	2.42 ± 0.14 ^d^	5.90 ± 0.35 ^b,c^
Wax	12.53 ± 0.19 ^a^	12.56 ± 2.61 ^a^	15.07 ± 1.27 ^a^	4.17 ± 0.62 ^b^
Balsam	44.43 ± 2.83	55.00 ± 8.66	51.67 ± 2.89	48.33 ± 3.54

* *n* ≥ 3, except for G19 (*n* = 2) and for the ash content of G20 (*n* = 2) due to a sample shortage. ^a, b, c, d^—differences considered statistically significant (*p* < 0.05) were noted with different letters.

**Table 2:** Total polyphenols content (TPC), total flavonoid content (TFC) and total ortho-diphenol content (TOC) determined for the four prepared G.EEs. The results are presented as mean ± SD (*n* ≥ 3). Different letters mean statistically significant differences between values (*p <* 0.05).

Parameters	G19.EE	G20.EE	G21.EE	G22.EE
TPC (mg GAE/g extract)	135.2 ± 8.2 ^b^	226.73 ± 4.3 ^a^	136.33 ± 2.23 ^b^	107.96 ± 5.6 ^c^
TFC (mg QE/g extract)	31.9 ± 1.1 ^c^	36.88 ± 0.5 ^b^	40.94 ± 1.32 ^a^	39.26 ± 0.9 ^a,b^
TOC (mg GAE/g extract)	272.3 ± 4.7 ^b^	290.46 ± 16.7 ^b^	332.27 ± 9.58 ^a^	292.24 ± 12.9 ^b^

^a, b, c^—differences considered statistically significant (*p* < 0.05) were noted with different letters.

**Table 3:** DPPH• and ABTS• scavenging activities of the four prepared propolis extracts (G19.EE–G22.EE). Results are expressed in EC_50_ (μg/mL) as mean ± SD (*n* ≥ 3). Gallic acid and trolox were used as standards for the DPPH and ABTS assays, respectively.

Free-Radical Scavenging Activity EC_50_ (µg/mL)	G19.EE	G20.EE	G21.EE	G22.EE	Gallic Acid	Trolox
DPPH•	17.19 ± 0.10 ^b^	21.49 ± 1.15 ^a^	10.81 ± 0.18 ^d^	13.37 ± 0.38 ^c^	1.21 ± 0.08	-------
ABTS•	10.53 ± 0.18 ^a^	10.11 ± 0.30 ^a^	9.36 ± 0.45 ^b^	8.38 ± 0.24 ^c^	--------	3.46 ± 0.22

^a, b, c, d^—differences considered statistically significant (*p* < 0.05) were noted with different letters.

**Table 4:** Minimum inhibitory concentration (MIC) values (µg/mL) of the ethanol extracts of Gerês propolis harvested from 2019 until 2022, against the panel of selected susceptibility indicator microorganisms.

		MIC (µg/mL)			
Strains		G19.EE	G20.EE	G21.EE	G22.EE
Yeast	*Candida albicans*	1500	>2000	2000	2000
	*Saccharomyces cerevisiae*	>2000	>2000	2000	2000
Bacteria	Gram-positive				
	*Bacillus cereus*	100	100	50	50
	*Bacillus megaterium*	100	100	100	50
	*Bacillus subtilis*	100	100	50	50
	*Propionibacterium acnes*	500	>2000	200	200
	MSSA *	500	750	200	200
	MRSA **	>2000	>2000	>2000	>2000
	Gram-negative				
	*Escherichia coli*	>2000	>2000	>2000	>2000

* MSSA = Methicillin-sensitive *Staphylococcus aureus*; ** MRSA = Methicillin-resistant *Staphylococcus aureus.*

## Data Availability

Data are contained within the article.
